# Functional outcomes and quality of life after surgically treated tibial plateau fractures

**DOI:** 10.1186/s40359-023-01195-2

**Published:** 2023-05-03

**Authors:** Abolfazl Bagherifard, Seyed Farzam Mirkamali, Heeva Rashidi, Nima Naderi, Mohammad Hassanzadeh, Mehdi Mohammadpour

**Affiliations:** Bone and Joint Reconstruction Research Center, Department of Orthopedics, School of Medicine, Iran University of Medical Sciences, Tehran, Iran

**Keywords:** Tibial plateau fracture, Patient-reported outcome measures, Quality of life, Surveys and questionnaires

## Abstract

**Background:**

Tibial plateau fractures (TPF) are uncommon and challenging for orthopedic surgeons with controversial reported outcomes. In this study, we aimed to evaluate the functional outcomes and quality of life (QOL) of patients with surgically treated TPF.

**Methods:**

A total of 80 consecutive patients and 82 controls participated in this case control study. The patients were all surgically treated in our tertiary center from April 2012 to April 2020. The functional outcome was evaluated using the Western Ontario and McMaster Universities Arthritis Index (WOMAC) scale. Moreover, we used the Short Form 36 health survey (SF-36) health survey to evaluate the QOL.

**Results:**

No significant difference was observed in the overall mean SF-36 score in the two groups. We found a significant positive correlation between the scores of the SF-36 and WOMAC questionnaires (*r* = 0.642, *p* < 0.001) and between the ROM and the WOMAC questionnaire score (r = 0.478, *p* < 0.001). Further, ROM and SF-36 showed a weak positive correlation (*r* = 0.248, *p* = 0.026). Age had a weak negative correlation with the pain subscale of SF-36 (*r* = − 0.255, *p* = 0.22), even though it was not correlated with the total score or other subscales (*p* > 0.05).

**Conclusion:**

QoL after TPF is not significantly different from that of a matched control group. Also, neither age nor BMI correlates with the QoL and functional outcome.

## Introduction

With an incidence rate of 10.3 per 100,000 people annually and 1% of all fractures, TPF is not uncommon [1–5]. TPF usually occurs in young patients and after high-energy traumas [6, 7]. These fractures are associated with a high rate of complications, including soft tissue injury, nerve damage, vascular injury, infection, delayed union, nonunion, and osteoarthritis (OA), which makes them one of the most challenging injuries to treat [7–10]. Also, due to their intraarticular nature, it is of utmost importance to achieve anatomical reduction and limb alignment [5, 8, 11].

Several treatment methods have been used and suggested, including closed reduction and casting, open reduction and internal fixation (ORIF), external fixation, or a combination of these methods [7]. The most common treatment method for TPF is ORIF performed by plates and screws, which has been associated with acceptable clinical results. However, this method is associated with complications, the most important of which are: excessive bone damage and soft tissue damage, high risk of infection, and functional rehabilitation problems with delayed activity and scar formation [12, 13]. Another treatment method for these patients is closed reduction and plastering, which is not recommended for adults due to the difficulty of maintaining the reduction in plaster and the possibility of complications such as malunion. Also, considering that this method requires a long period of immobility, it can lead to knee stiffness, and this method is mostly used for patients who are not medically eligible for surgery [14–16]. Hybrid treatment can be the most ideal treatment method for these patients. In this method, it can be used in compound injuries as well as in fractures with extensive soft tissue damage as a definitive fixation option, in which the hybrid external fixator combines an Ilizarov ring with a standard AO frame [14, 17]. Also, with the use of new devices, the techniques have improved significantly [18]. However, the complication rate still remains high, and these fractures are associated with poor prognosis as up to 58% of the patients develop OA [7, 19–22]. Because fractures in this area are associated with several complex injuries such as soft tissue damage, deep abrasions, combined injuries and blister formation due to extensive edema and crushing at the fracture site along with ligament injuries that lead to instability and often it is difficult to manage these patients and the postoperative complications will still be high in these patients [14].

This type of fracture and its surgical management remains a challenge due to its complexity [23]. Also, several studies which evaluated functional outcomes and quality of life (QoL) after TPF and OA have reported unsatisfactory results [23, 24]. Moreover, the proximal tibia fractures resulted in worse outcomes than outcomes of midshaft or distal tibial fractures [25]. However, to the best of our knowledge, no study compared the results of their evaluation against a matched control group. In this study, we aimed to investigate the patient-reported functional outcomes and QoL of the patients after surgical treatment of TPF and compare the QoL with a matched control group. The aim of this study was to answer two questions: 1. What is the functional outcome in patients with TPF and 2. Will the quality of life of patients after surgery be different compared to the normal and healthy group or not?

## Material and methods

### Participants

This prospective case–control study took place in Shafa Yahyaian hospital, Tehran, Iran, from April 2012 to April 2020. All consecutive patients with TPF presenting to our hospital, either through the emergency department or clinic, were included in the study. Sampling of patients was done as available and among all referring patients. The institutional review board and the ethics committee of Iran University of Medical Sciences approved this study. All participants provided verbal informed consent.

### Inclusion and exclusion criteria

The inclusion criteria were a diagnosis of TPF, no fractures in the other knee or spine, no history of knee surgery, cooperation for follow-up, and at least six months of follow-up after surgery. The exclusion criteria were history of any previous surgery on the lower limbs that affects the outcome of treatment, less than six months of follow-up, defects in clinical and radiological finding or demographic information, lack of cooperation or reluctance to participate in the study and less than 18 years of age.

### Study groups

A matched population of 82 subjects with no history of TPF or any other pathology in the knee or fibula was enrolled from the community as the control group. Sampling of the control group was done randomly and from healthy people who referred to the hospital. The groups were matched in terms of age, gender, and BMI of the patients. Patients' information was collected in two parts using a two-part checklist. Demographic and clinical data and follow-up time were recorded. All patients were followed up and visited after the operation to evaluate the operation outcomes, QoL, and postoperative functional results.

### Outcome measurement instruments

We used the WOMAC scale to evaluate patient-reported outcomes, which was used after these fractures [7, 26, 27]. The WOMAC questionnaire is scored on a 0–100 scale and consists of 25 items in 5 domains which are answered by a Likert scale and evaluates pain, clinical symptoms and stiffness, daily function, exercise, and QoL [28]. A higher score on this scale shows inferior outcomes. This scale has been widely approved for the Iranian population in terms of reliability and validity [13, 14].

Furthermore, QoL was assessed using the SF-36 health survey, which is an established method in patients with this type of fracture [23, 29], and has been validated in Persian [30]. SF-36 consists of 36 items in 8 subscales, including pain, energy/fatigue, role limitations due to physical health, physical functioning, role limitations due to emotional problems, emotional wellbeing, social functioning, and general health. The score in each subscale and the total score were converted to a 0–100 scale, in which a higher score indicates a better status [31].

### Statistical analysis

We used SPSS version 25.0 to analyze data. The Chi-Square test was used to evaluate the relationship between the categorical variables. We used the Kolmogorov–Smirnov method to check the distribution of the data. To compare the quantitative variable between two groups, independent samples t-test was used. The Mann–Whitney *U* test was considered a suitable substitute if the data lacked the parametric criteria. Spearman and Pearson's tests were used to assess correlation. A *p* value less than 0.05 was considered significant for statistical tests.

## Results

Overall, 162 participants (80 cases and 82 controls) were enrolled in the study. Age and BMI were comparable between the groups (Table 1).

**Table 1:** Demographic characteristics in two groups

Variable	Group			*p* value
	Fracture (n = 80)	Control (n = 82)	Total	
Age^a^	45.39 (13.31)	44.27 (12.78)	44.82 (13.01)	0.586
BMI^b^	27.49 (4.95)	26.63 (4.65)	27.05 (4.81)	0.152

^a^*t* test; ^b^Mann–Whitney U test data presented as mean (SD)

The total mean WOMAC score in patient was 77.83 ± 16.81 (Table 2). The patients were followed up for 48.20 ± 22.43 months. Also, comorbidities were comparable in the groups (*p* value = 0.651). No significant difference was observed for the mean score of SF-36 and its subscales in the two groups (Table 3).

**Table 2:** Mean WOMAC score and subscales in patient group

Variable	Mean (SD)
WOMAC	77.83 (16.81)
Pain	82.91 (18.03)
Clinical symptoms and stiffness	85.93 (15.64)
Daily function	85.27 (15.64)
Exercise	64.69 (24.94)
Quality of life	70.50 (21.41)

**Table 3:** Comparison of mean SF-36 score and subscales in two groups of patients

Variable	Group			*p* value
	Case (n = 80)	Control (n = 82)	Total	
SF-36^2^	79.78 (15.46)	83.37 (11.70)	81.59 (13.77)	0.224
Pain	73.06 (22.53)	78.60 (17.12)	75.86 (20.13)	0.169
Energy/fatigue	87.79 (23.02)	94.15 (13.02)	91.01 (18.85)	0.509
Role limitations due to physical health	81.73 (25.70)	85.33 (23.12)	83.55 (24.42)	0.846
Physical functioning	76.10 (18.19)	76.23 (18.45)	76.17 (18.27)	0.256
Role limitations due to emotional problems	79.59 (16.33)	82.12 (15.94)	80.87 (16.13)	0.141
Emotional wellbeing	81.29 (19.93)	84.95 (19.27)	83.15 (19.62)	0.094
Social functioning	83.37 (18.71)	88.55 (14.29)	85.99 (16.76)	0.523
General health	75.33 (18.96)	77.05 (18.31)	76.20 (18.59)	0.326

We also found a significant positive correlation between the scores of the SF-36 and WOMAC questionnaires (*r* = 0.642, *p* < 0.001) and between the ROM and the WOMAC questionnaire score (*r* = 0.478, *p* < 0.001). Further, ROM and SF-36 showed a weak positive correlation (*r* = 0.248, *p* = 0.026). BMI was not significantly correlated with the WOMAC or SF-36 scores or any of the subscales (*p* > 0.05). Also, age was not significantly correlated with the WOMAC score or any of its subscales (*p* > 0.05). Age had a weak negative correlation with the pain subscale of SF-36 (*r* = − 0.255, *p* = 0.22), even though it was not correlated with the total score or other subscales (*p* > 0.05).

## Discussion

In this study, we evaluated the patient-reported functional outcome using the WOMAC questionnaire and QoL using the SF-36 questionnaire and after TPF and compared QoL with a matched control group. The QoL in patients was not significantly different from the control group, either in the total score or in any specific items. We found no correlation between age and ROM, the WOMAC score, or any of its subscales; however, we found a weak negative correlation between age and pain subscale of SF-36. Furthermore, BMI had no correlation with ROM, the WOMAC or SF-36 scores, or any subscales.

Elsøe et al. Evaluated QoL, functional and radiological outcomes in 28 patients with lateral TPF. They showed that patients had a high level of satisfaction and their functional outcomes were not significantly different from the reference population [26]. These results were consistent with our study, which shows that patients with PTF regain their functional ability after surgery as before the fracture. These findings show the quality of life of patients with TPF after surgery, which consists of different dimensions such as pain, energy/fatigue, role limitations due to physical health, physical functioning, role limitations due to emotional problems, emotional well-being, social functioning and general health, was not different in the case and control groups.

As already mentioned, TPF can result in OA [3, 32]. Vandreumel et al.,(7) Assessed 71 consecutive patients with TPF to evaluate mid- to long-term functional outcomes after surgical treatment of TPF. In their study, they did not report a relationship between mean WOMAC score and age for any subscale. These findings align with our results that age and BMI do not affect the WOMAC score. These findings show that TPF surgery significantly improves functional outcomes (WOMAC score and SF-36 score) of patients, regardless of the demographic characteristics of patients (such as BMI and age). The reason for the difference between this finding in our study and other studies can be justified due to the fact that the case and control groups are identical (with the aim of controlling confounders. Another study reported that treating TPF results in 73.3% excellent to good functional outcomes and satisfactory QoL assessment [18]. This report is concordant with our findings.

The fact that the literature regarding the functional outcome of surgically treated TPF is controversial may be due to different inclusion criteria, various classification systems and fixation techniques, or missing functional outcome data [33].

Our studies had weak and strong points that should be pointed out. The first limitation of the study is the retrospective design which causes known limitations. Also, in this study, we did not report the type of fracture and treatment method, which could be noteworthy. Moreover, the patients were followed for less than ten years; thus, we could not report long-term outcomes.

## Conclusion

Our study showed, the TPF although challenging, do not necessarily result in inferior QoL. As we found that the QoL of patients after surgical treatment of TPF was not significantly different from that of a matched control group. Also, neither age nor BMI correlates with the QoL and functional outcomes as measured by SF-36 and WOMAC questionnaires.

## Data Availability

Given that the data of this study is a small part of the data of a large study and according to the forecast of a series of articles will be extracted serially from this data. Data will not be available until the end of the project, although the datasets used and analyzed during the current study are available from the corresponding author upon reasonable request.

## Abbreviations

BMI: Body mass index
WOMAC: Western Ontario and McMaster Universities Arthritis Index
TPF: Tibial plateau fractures
QoL: Quality of life
SF-36: Short Form 36 health survey
